# Muscle Transcriptome Analysis Reveals Molecular Pathways Related to Oxidative Phosphorylation, Antioxidant Defense, Fatness and Growth in Mangalitsa and Moravka Pigs

**DOI:** 10.3390/ani11030844

**Published:** 2021-03-16

**Authors:** Yolanda Núñez, Čedomir Radović, Radomir Savić, Juan M. García-Casco, Marjeta Čandek-Potokar, Rita Benítez, Dragan Radojković, Miloš Lukić, Marija Gogić, María Muñoz, Luca Fontanesi, Cristina Óvilo

**Affiliations:** 1 Departamento de Mejora Genética Animal, Instituto Nacional de Investigación y Tecnología Agraria y Alimentaria (INIA), 28040 Madrid, Spain; nunez.yolanda@inia.es (Y.N.); garcia.juan@inia.es (J.M.G.-C.); rmbenitez@inia.es (R.B.); mariamm@inia.es (M.M.); 2 Institute for Animal Husbandry, 11080 Belgrade, Serbia; cedomirradovic.izs@gmail.com (Č.R.); miloslukic.izs@gmail.com (M.L.); gogic.marija@gmail.com (M.G.); 3 Faculty of Agriculture, University of Belgrade, 11080 Belgrade, Serbia; savic@agrif.bg.ac.rs (R.S.); radodrag@agrif.bg.ac.rs (D.R.); 4 Agricultural Institute of Slovenia (KIS), 1000 Ljubljana, Slovenia; meta.candek-potokar@kis.si; 5 Department of Agricultural and Food Sciences, University of Bologna, 40126 Bologna, Italy; luca.fontanesi@unibo.it

**Keywords:** transcriptomics, nutrigenomics, pig, muscle, Mangalitsa, Moravka, tannins

## Abstract

**Simple Summary:**

The study of gene expression at the transcriptome level provides rich information on the genes and metabolic pathways influencing relevant phenotypic traits. This study deals with the comparison of muscle transcriptome between two autochthonous pig breeds from Serbia (Mangalitsa and Moravka) as well as between Mangalitsa pigs fed a tannin-supplemented diet vs. a control one. The results provide a wide characterization of genes, pathways and potential regulatory mechanisms affecting muscle traits which differ among the experimental groups. The generated information improves the scientific knowledge on the cellular and metabolic basis of muscle growth, oxidative stability and fatness in pigs, and provides abundant candidate genes which could be responsible for phenotypic variation, and could be useful in future studies and selection approaches.

**Abstract:**

This work was aimed at evaluating loin transcriptome and metabolic pathway differences between the two main Serbian local pig breeds with divergent characteristics regarding muscle growth and fatness, as well as exploring nutrigenomic effects of tannin supplementation in Mangalitsa (MA) pigs. The study comprised 24 Mangalitsa and 10 Moravka (MO) males, which were kept under identical management conditions. Mangalitsa animals were divided in two nutritional groups (n = 12) receiving a standard (control) or tannin–supplemented diet (1.5%; MAT). Moravka pigs were fed the standard mixture. All animals were slaughtered at a similar age; 120 kg of average live weight (LW) and loin tissue was used for RNA-seq analysis. Results showed 306 differentially expressed genes (DEGs) according to breed, enriched in genes involved in growth, lipid metabolism, protein metabolism and muscle development, such as *PDK4*, *FABP4*, *MYOD1* and *STAT3*, as well as a relevant number of genes involved in mitochondrial respiratory activity (*MT-NDs*, *NDUFAs* among others). Oxidative phosphorylation was the most significantly affected pathway, activated in Mangalitsa muscle, revealing the basis of a different muscle metabolism. Also, many other relevant pathways were affected by breed and involved in oxidative stress response, fat accumulation and development of skeletal muscle. Results also allowed the identification of potential regulators and causal networks such as those controlled by *FLCN*, *PPARGC1A* or *PRKAB1* with relevant regulatory roles on DEGs involved in mitochondrial and lipid metabolism, or *IL3* and *TRAF2* potentially controlling DEGs involved in muscle development. The Tannin effect on transcriptome was small, with only 23 DEGs, but included interesting ones involved in lipid deposition such as *PPARGC1B*. The results indicate a significant effect of the breed on muscle tissue gene expression, affecting relevant biological pathways and allowing the identification of strong regulatory candidate genes to underlie the gene expression and phenotypic differences between the compared groups.

## 1. Introduction

Pork is one of the most widely consumed meats in the world. In western countries, pig production mostly relies on a few commercial breeds subjected to intensive breeding practices, but also on numerous autochthonous breeds, which are usually well adapted to the environment and suitable for outdoor and organic production and for manufacturing of traditional pork meat products. Mangalitsa and Moravka are the two main indigenous pig breeds raised in the Republic of Serbia, the first one being the most widespread [1,2], and present not only in Serbia but also in other European countries. In recent years, the interest in the study of native autochthonous breeds has risen due to the relevance of genetic resource preservation, the production of traditional meat products and the maintenance of sustainable local pork chains. Swallow-belly Mangalitsa, the most abundant strain in Serbia, is considered a fatty-type breed, with 65–70% of carcass fat content and less than 40% of lean meat in carcass sides [1,3]. Its meat is characterized by high and variable intramuscular fat content (ranging from 2.9 to 18.2% in different studies [1], low water content, intensive red color and firm consistency, and is used for the production of high quality meat products such as ham and sausages. In contrast, Moravka is a breed with combined production abilities, characterized by a higher meat percentage and longer carcass with less fat, usually also in meat, than Mangalitsa [4]. Regarding their genetic origin the Moravka breed is an admixed population [5,6] which was created by unsystematic crossings of the extinct indigenous breed Šumadinka with Berkshire and possibly with Yorkshire [2], while the Mangalitsa breed is also derived from the primitive Šumadinka but without crossing with improved genotypes and without Asian introgression [1,6].

Genetic background and nutrition are the main factors affecting animal performance and consequently tissue composition. Although tannins are in general considered anti-nutritive substances, tannin supplementation of the diet for animals has mainly been studied because of its antimicrobial, antihelmintic or antioxidant properties [7]. Indeed, chestnut wood extract, which is a source of hydrolysable tannins, has been proposed to reduce oxidative stress in pigs and poultry [8] and to increase the oxidative stability of meat [9]. Nevertheless, hydrolysable tannins have been shown to reduce protein digestibility [10], so their supplementation could affect growth performance. Thus, the dosages in the majority of the studies are usually low, although somewhat higher dosages have been tested in studies on boar taint aimed at reducing intestinal production of skatole [11,12,13,14,15]. For better utilization of local feedstuffs, more knowledge on the molecular mechanisms influencing growth or meat quality differences under different dietary regimes is needed. Effects of bioactive compounds on tissue metabolism and phenotype are expected to be mediated, at least partially, by changes in tissue gene expression, as reported in nutrigenomic studies [16]. Although the effects of tannins supplementation has been explored at the level of microbiota composition and intestine and hepatic candidate gene expression [12,14], with a focus on boar taint compounds metabolism, to the best of our knowledge there is no previous work exploring nutrigenomic effects of chestnut wood extract-supplemented diets on pig muscle.

Recently, advances in high-throughput omics technologies and combination with nutrition have allowed us to better understand the complex biology behind economically important traits. Novel approaches are being used to discover gene networks associated with particular phenotypes. Previous works compared muscle transcriptome of different pig genotypes and identified genes and regulatory networks associated with growth, fatness and metabolism [17,18,19,20], although Mangalitsa and Moravka transcriptomes have not been explored so far.

Mangalitsa (swallow-belly strain) and Moravka were included in the EU research and innovation project TREASURE (www.treasure.kis.si; accessed on 13 March 2021), which dealt with traditional genetic resources and the study of their potential and molecular basis for growth as well as the quality of their products. The present study was designed with the objectives of evaluating, in the *longissimus dorsi* muscle, the whole transcriptomic differences between the two main Serbian local pig breeds with divergent characteristics regarding muscle growth and fat accretion, as well as exploring the pathways in which the differentially expressed genes are involved and the potential regulators of the gene expression differences. Also, nutrigenomic effects of tannin supplementation were explored on muscle transcriptome of Mangalitsa pigs.

## 2. Materials and Methods

### 2.1. Ethics Statement

The experiment was conducted with the approval of the Trial Animals Welfare Ethical Council of the Veterinary Directorate (Decision no. 323-07-10545/2015-05/2) of the Ministry of Agriculture, Forestry and Water Management, Republic of Serbia. 

### 2.2. Animals and Sampling

The trial was carried out in the facilities of the Institute for Animal Husbandry, Zemun-Belgrade, with castrated males of Mangalitsa (n = 24) and Moravka (n = 10) breeds. Piglets were purchased from individual breeding farms and delivered immediately after weaning. The animals were kept in facilities with semi-outdoor system. The surface of outdoor area was 150 m^2^ for each pen (110 m^2^ open and 40 m^2^ covered part). Each experimental group was housed in a separate pen with outdoor area, and had a separate feeder. Floor/feeder space was wide enough for avoiding competition and growth differences due to the unequal number of animals within the groups (10 for Moravka and 12 for Mangalitsa).

At about 22 kg live weight (LW) and 156 days of age, the piglets were assigned to three experimental groups in separate sections: Mangalitsa control (MA, n = 12), Mangalitsa treated with tannins (MAT, n = 12) and Moravka (MO, n = 10). Complete feed mixtures (Table 1) were used for both breeds, and pigs were fed ad libitum with tube machines (capacity 110 kg). From 25 to 60 kg LW, mixture I was used, including: 15% of crude protein (CP) and 13.6 MJ ME/kg. From 60 to 120 kg LW mixture II was provided, with 13% CP and 13.5 MJ ME/kg. In the mixture for the MAT group, chestnut wood extract (2%) was included instead of the corresponding amount of corn. Chestnut wood extract contains 75% hydrolysable tannins, meaning 1.5% tannin supplementation in the complete mixture (15 g of hydrolysable tannins per kg). This product was purchased from the company Tanin Sevnica d.d. (Sevnica, Slovenia; http://www.tanin.si/; accessed on 13 March 2021). Weighing of pigs was done at the start and end of the experiment and the amount of feed consumption was also recorded at the group level. The average daily gain was individually calculated for each pig. Pigs had free access to water and were fed throughout the experiment an average of 2.4 (MA and MAT group) and 2.6 kg/day (MO group) of complete mixture per animal (measured at group level). 

The trial lasted from March to August. When reaching the target slaughter weight (120 kg), the pigs were weighed and transported to an experimental slaughterhouse and slaughtered at a similar age. 

### 2.3. Phenotypic Analysis

The research encompassed the monitoring of individual weights until slaughter (kg) and calculated average live daily gain (g). Traits measured on the slaughter line included carcass weight (kg) and thickness (mm) of *longissimus dorsi* muscle (from the end of spinal column to the cranial part of *gluteus medius* muscle). 

*Longissimus dorsi* muscle samples (300 g) were collected, marked and frozen (−20 °C) before chemical analyses, which were done in the reference laboratory of the Institute of Meat Hygiene and Technology in Belgrade. Prior to laboratory analysis, muscle samples were defrosted, cut into pieces and homogenized in a blender CombiMax600. Intramuscular fat content (IMF, %) was determined with the extraction method by Soxhlet. Fatty acids as methyl esters were determined using the capillary gas chromatography with flame-ionization detector. Fatty acids were converted into fatty acid methyl esters (FAME) with tri-methyl sulphonium hydroxide according to the SRPS EN ISO 5509:2007 method. Fatty acid methyl esters were analyzed on the apparatus GC-FID Shimadzu 2010 (Shimadzu, Kyoto, Japan) on cyanopropyl-aryl column HP-88. A volume of 1 µL was injected. Injector and detector temperatures were 250 °C and 280 °C, respectively. Nitrogen was used as the carrier gas and hydrogen and air as detector gases. The column furnace temperature was programmed in the range of 120 °C to 230 °C. Fatty acid methyl esters were identified based on the retention time, by comparing the retention times of the mixture of fatty acid methyl esters in the standard, Supelco 37 Component FAME Mix [21]. Percentage of each fatty acid to the total of fatty acids was determined. Statistical analysis of breed and tannin effects on phenotypic traits was carried out by applying the General Linear Model procedure of the statistical package SAS 9.1.3 (SAS Inst. Inc., Cary, NC, USA, 2002–2003) using the model:

y_ij_ = μ + G_i_ + e_ij_,

where: y_ij_ is the analyzed trait; μ is overall mean; G_i_ is the effect of group (MA vs. MO / MA vs. MAT); and e_ij_ is a random error. The statistical significance of differences between least-squares means (LS Means) was determined by t-test. The *p* values lower than 0.05 were considered significant while values in the range 0.05–0.10 were considered as tendencies. The pig was considered the experimental unit in the study.

### 2.4. Transcriptome Analysis

#### 2.4.1. RNA Extraction

Samples from *longissimus dorsi* muscle were collected from each animal and stored in RNAlater (Qiagen, Redwood City, CA, USA; www.qiagen.com; accessed on 13 March 2021) for transcriptome analysis. Fifteen animals were used for the transcriptomic study, 5 animals of each experimental group: Mangalitsa control (MA), Mangalitsa treated with tannins (MAT) and Moravka (MO). Total RNA was isolated from 100 mg samples of *longissimus dorsi* muscle using the RiboPureTM RNA isolation kit (Ambion, Austin, TX, USA), following the manufacturer’s recommendations. The obtained RNA was quantified using NanoDrop equipment (NanoDrop Technologies, Wilmington, DE, USA) and the RNA quality was assessed with an Agilent 2100 bioanalyzer device (Agilent Technologies, Palo Alto, CA, USA) with resulting RNA Integrity Number (RIN) values greater than 8. RNAs were submitted to the CNAG_CRG (Centro Nacional de Análisis Genómico, Barcelona Spain) for high throughput sequencing. 

#### 2.4.2. RNA Library Construction and Sequencing

RNA libraries were constructed using the mRNA-seq sample preparation kit (Illumina, San Diego, CA, USA) with standard protocols. Libraries were sequenced using a TruSeq SBS Kit v3-HS (Illumina) in paired-end stranded mode with a read length of 2 × 76 bp on a HiSeq2000 sequence analyzer (Illumina).

#### 2.4.3. Mapping, Assembly and Differential Expression Analysis

FastQC software (Babraham Institute, Cambridge, UK; www.bioinformatics.babraham.ac.uk/projects/fastqc/; accessed on 13 March 2021) was used for the quality control of the raw sequences. All the samples passed the quality control parameters based on read length (76 bp), 100% coverage in all bases, A, T, G and C nucleotide contributions close to 25%, 40–60% GC content and less than 0.1% overrepresented sequences. Adaptor sequences, poli (A)/poli (T) tails and reads shorter than 40 bp were removed from the dataset using Trim Galore software (version 0.4.1, Babraham Institute, Cambridge, UK; https://www.bioinformatics.babraham.ac.uk/projects/trim_galore/; accessed on 13 March 2021). Processed reads were mapped against the pig reference genome (Sscrofa11.1) using HISAT2 [22]. Resulting SAM files were then converted to BAM files with Samtools 1.9. (Wellcome Trust Sanger Institute, Hinxton, UK; https://www.sanger.ac.uk/tool/samtools-bcftools-htslib/; accessed on 13 March 2021)

Raw counts were generated for each gene locus using HTSeq package (Python Software Foundation, Beaverton, OR, USA; https://pypi.python.org/pypi/HTSeq; accessed on 13 March 2021). Differential expression analysis was performed with the DESeq package [23]. Gene-based expression matrix was used to compare both breeds (MA vs MO) and the tannin treatment within the Mangalitsa breed (MA vs. MAT). Genes were considered differentially expressed (DEG) when they were DE according to the criteria: *p*-adjusted value (*q*) ≤ 0.10 and Fold Change (FC) > 1.5.

#### 2.4.4. Reverse Transcription Quantitative PCR (RT-qPCR)

RNA obtained from the 15 animals included in the RNA-seq assay was used to perform the technical validation of some DEGs. Primers were designed against each gene, covering different exons to assure the amplification of the cDNA, using Primer Select Software (DNASTAR, Madison, WI, USA) from the available ENSEMBL sequences. The specific primer details are shown in Table S1.

First strand cDNA synthesis was carried out with SuperScript II (Invitrogen, Life Technologies, Paisley, UK) and random hexamers in a total volume of 20 µL containing 1 µg of total RNA and following the supplier’s instructions.

In the breed comparison study (Mangalitsa vs. Moravka), validation was carried out on nine genes using *GAPDH* and *ACTB* as housekeeping genes: *MT-ND2, ATP6, COX2, PDK4, JAZF1, NOS1, MYOD1* and *STAT3* were selected as DEGs, and *FOS* was analyzed as non-DEG. In the diet comparison (MA vs. MAT) validation was carried out on 6 genes with *ACTB* and *B2M* being used as housekeeping genes: *ARID5A, DAPK3, TNFRSF12A* and *PPARGC1B* were selected as DEGs and *RUNX1*, *ACLY* as non-DEGs. The selection of the two most stable endogenous genes for data normalization in each comparison was performed by evaluating *GAPDH, ACTB, TBP, 18S, PPIA* and *B2M* with the Genorm and the Normfinder softwares [24,25]. Transcript quantification was performed using SYBR green mix (Roche, Rotkreuz, Switzerland) in a LightCycler 480 II (Roche, Rotkreuz, Switzerland).

#### 2.4.5. Statistical Analysis of qPCR Data

The method proposed by Steibel et al. [26] was used for the statistical analysis of qPCR gene expression data. This procedure simultaneously analyzes the Crossing point (Cp) values for the target and endogenous genes using a linear mixed model, which included the effects of the breed (two levels: MA and MO) or the diet (two levels: MA and MAT). Pearson’s correlation was used to compare counts values obtained from RNA-seq analysis with the Genorm-normalized gene expression data obtained by RT-qPCR. The concordance correlation coefficient (CCC) [27] was calculated between the FC values estimated from RNA-seq and qPCR expression measures for the genes analyzed by both technologies.

#### 2.4.6. Functional Analysis

Differentially expressed genes were functionally analyzed using Ingenuity Pathway Analysis software (IPA, QIAGEN Redwood City, CA, USA; www.qiagen.como/ingenuity; accessed on 13 March 2021) to detect enriched pathways, biological functions and potential regulators.

## 3. Results and Discussion

Mangalitsa and Moravka, the two main autochthonous pig breeds raised in Republic of Serbia, differing phenotypically and genetically [1,2,5,6], have been scarcely explored at the molecular or genetic levels. To the best of our knowledge there is no transcriptome nor nutrigenomic study aimed at analyzing the functional genetic basis of muscle physiology in these breeds. In the present work, muscle tissue samples obtained from purebred Mangalitsa and Moravka breeds as well as tannin-fed Mangalitsa pigs were used for transcriptomic analysis by RNA-seq, in order to better understand the effects of breed and diet on *longissimus dorsi* muscle metabolism.

### 3.1. Validation by RT-qPCR 

In order to validate the results obtained in the RNA-seq analysis, the relative expression of a selection of genes was quantified using Real-time-qPCR, including some DEG and a few non-DEG. Pearson correlation was calculated between the expression values obtained from RNA-seq data (counts) and normalized gene expression data obtained by RT-qPCR, with significant results for all the tested genes (correlation between 0.626 and 0.999) (Table 2). Also, the concordance correlation coefficient (CCC) used to assess technical validation in high throughput transcriptomic studies [27] denoted an acceptable concordance between RNA-seq and qPCR expression values (CCC = 0.72). Fold-Change and significance tended to be greater when expression differences were analyzed by RNA-seq technology, in accordance with its higher sensitivity.

### 3.2. Breed and Diet Effects on Studied Phenotype

Phenotypic results are shown in Table 3. Data on growth rates of the Mangalitsa and Moravka populations employed in this study have been reported in-depth elsewhere [28]. In brief, Moravka pigs exhibited a higher average growth rate than Mangalitsa pigs, Moravka pigs being 13% heavier than Mangalitsa pigs at the end of the study, with similar age at slaughter (357 vs. 363 days). Results corresponding to the 34 animals used in the present study (Table 3) are in agreement with those at the population level, with higher average daily gain, slaughter weight and carcass weight observed in Moravka [1,2]. Regarding muscle development, slightly higher *l. dorsi* thickness (*p* = 0.08) is observed in Moravka, in agreement with its higher growth potential. Regarding muscle composition, a few significant findings can be observed concerning differences in the percentages of several fatty acids, such as higher palmitoleic (C16:1) and lower stearic acid (C18:0) in Mangalitsa, and higher eicosenoic (C20:1) in Moravka, but no statistically significant difference in intramuscular fat content is detected. Although in general Mangalitsa are considered fatter than Moravka pigs, and different works have reported such difference [4,29,30], there are also studies reporting the opposite trend in the content of fat in loin muscle [31]. According to our results there is a numerical difference matching the previously reported higher fattening trend and meat quality in Mangalitsa [4]. This difference would possibly be increased if the comparison was made with weight-paired, instead of age-paired, experimental groups.

Tannin supplementation didn’t have an effect on growth and carcass traits, but significantly higher intramuscular fat content was detected in MAT group, with a strong effect on this trait (+3.11%; *p* = 0.04). When it comes to muscle quality, the tannin supplemented group showed lower content of erucic and arachidonic (C22:1 + C20:4) unsaturated fatty acids (*p* = 0.016) and higher content of stearic acid (C18:0; *p* = 0.044). These results are mostly consistent with previous findings which already showed that supplementation of lean crossbred pig diets with chestnut wood extract had no detrimental effect on growth rate, carcass traits or meat quality ([32,33]), and may even show a beneficial effect on feed efficiency and intestinal health [34]. Prevolnik et al. [32] speculated that a tannin concentration of 2 g/kg was too low to cause detrimental effects. According to the present results a supplementation of 2% of chestnut wood extract (meaning 1.5% hydrolysable tannins) neither affect main growth and muscle development parameters in the fatty Mangalitsa breed, but show a positive effect on intramuscular fat content, which could favorably influence meat quality. 

### 3.3. Breed Effect on Transcriptome

Overall, sequencing quality was high with sequencing precision above 99%. An average of 41.6 million reads was obtained per sample. After trimming, an average of 39.3 million reads per sample remained and were used for subsequent analyses, thus 5.5% of reads were lost in the trimming step. GC content ranged between 54 and 57% in the different samples. Reads were mapped to porcine reference sequence Sscrofa11.1 obtaining over 97–98% alignment rate. A total of 14,213 genes from 22,452 genes annotated in the reference sequence were detected as being expressed in our samples. Comparison between muscle transcriptome of both breeds resulted in 306 differentially expressed genes (DEG), 180 overexpressed in Mangalitsa and 126 in Moravka. Fold change ranged from 1.5 to 11.5 in Mangalitsa and 1.5 to 45.6 in the Moravka breed. The complete list of DEGs is included in Table S2. 

Among the genes found upregulated in Mangalitsa there were, as expected, some candidate genes involved in lipid metabolism such as *PDK4* (Pyruvate Dehydrogenase Kinase 4, FC = 7.30 and *q* = 0.100) and *FABP4* (Fatty Acid Binding Protein 4, FC = 3.57 and *q* = 0.033). *PDK4* is a member of protein kinase family and encodes a mitochondrial protein. It is in charge of catalyzing phosphorylation / dephosphorylation of pyruvate dehydrogenase complex which is involved in the first step of glucose oxidation. Due to this role, *PDK4* contributes to the regulation of glucose and fatty acid metabolism (fatty acid oxidation and the novo fatty acid biosynthesis) [35]. It also plays a role in the generation of reactive oxygen species (ROS). In fact, the *PDK4* gene is considered a candidate for fattening traits and its structural variation has been associated with IMF, muscle water content (MWC) and other meat quality traits in pigs [36]. Fatty acid binding protein 4 (FABP4) encodes a cytoplasmic protein found in adipocytes which is in charge of long chain fatty acid binding and lipid transport. It is involved in the regulation of intramuscular fat accretion. In pigs, it is a recognized genetic marker for meat tenderness and IMF content [37].

**Figure 1:**
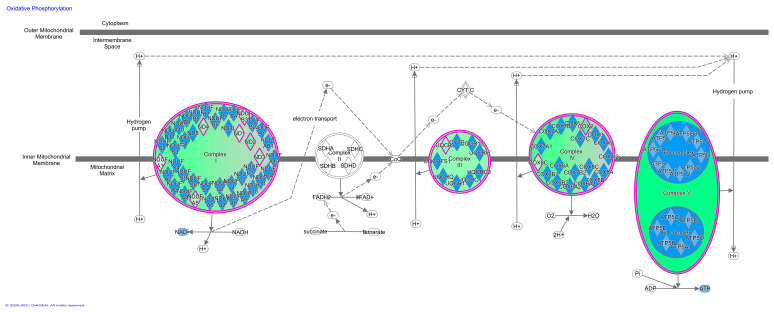
Oxidative phosphorylation pathway predicted to be activated in Mangalitsa, created using Ingenuity Pathway Analysis software (IPA).

Besides, an abundant group of genes involved in mitochondrial respiratory activity were observed to be upregulated in Mangalitsa. Skeletal muscle requires energy, both for use and storage, which is achieved from oxidation of glycogen, carbohydrates and fat, through oxidative phosphorylation. Oxidative phosphorylation is the process in which ATP is formed as a result of the transfer of electrons from NADH or FADH_2_ to O_2_ by a series of electron carriers. This pathway involves five protein enzyme complexes (complex I–V) located in the mitochondrial inner membrane. Twelve DEGs were found, all of them being upregulated in Mangalitsa breed, which are involved in 4 out of the 5 complexes (Figure 1).

Several of these DEGs upregulated in Mangalitsa belong to complex I, which transports electrons from NADH to ubiquinone, including *MT-ND1* (NADH-ubiquinone oxidoreductase chain 1; FC = 1.884; *q* = 0.109), *MT-ND2* (NADH-ubiquinone oxidoreductase chain 2; FC = 2.650; *q* = 0.019), *MT-ND3* (NADH-ubiquinone oxidoreductase chain 3, FC = 2.052; *q* = 0.075), *MT-ND4* (NADH-ubiquinone oxidoreductase chain 4; FC = 2.094; *q* = 0.037), *NDUFA1* (NADH-ubiquinone oxidoreductase subunit A1; FC = 1.609; *q* = 0.067), *NDUFA5* (NADH-ubiquinone oxidoreductase subunit A5; FC = 1.865; *q* = 0.097) and *NDUFB3* (NADH-ubiquinone oxidoreductase subunit B3; FC = 2.098; *q* = 0.003). No DEG belonging to complex II was found, which catalyzes the oxidation of succinate to fumarate and transfers electrons to the ubiquinone pool of the respiratory chain. In turn, another DE gene, *UQCRB* (ubiquinol-cytochrome c reductase binding protein, FC = 2.110; *q* = 0.003) belongs to complex III, which transfers electrons from ubiquinol to cytochrome c coupled with the transfer of electrons across the inner mitochondrial membrane. Complex IV, the final step in the electron transport chain, is the reduction of molecular oxygen by electrons derived from cytochrome C. Two DEGs belonging to this group were upregulated in Mangalitsa: *COX2* (Cytochrome c oxidase subunit II; FC = 2.823; *q* = 0.003) and *COX6C* (Cytochrome c oxidase subunit 6C; FC = 1.908; *q* = 0.025). Complex V, the final enzyme complex in the oxidative phosphorylation pathway, couples a proton gradient generated by respiratory chain to ATP synthesis where protons flow from intermembrane mitochondrial space to the matrix. Genes within this last complex were also altered between our breeds, with *ATP6* (Mitochondrially encoded ATP synthase membrane subunit 6; FC = 3.162; *q* = 0.001) and *ATP5MD* (ATP synthase membrane subunit DAPIT; FC = 1.932; *q* = 0.039) being upregulated in the Mangalitsa breed. Thus, there was a clear upregulation in Mangalitsa of the mitochondrial processes leading to energy production. 

An upregulation in Mangalitsa was also observed for several genes with roles in protein metabolism. Genes involved in protein synthesis within the mitochondria, such as *MRPL33* (Mitochondrial Ribosomal Protein L33; FC = 2.059; *q* = 0.012)), *MRPL42* (Mitochondrial Ribosomal Protein L42; FC = 2.159; *q* = 0.052) or *MRPS36* (Mitochondrial Ribosomal Protein S36; FC = 1.801; *q* = 0.097), were upregulated in Mangalitsa. Besides, different genes involved in protein catabolism processes were included in the DEG list, such as the chaperones *DNAJC2* (DnaJ Heat Shock Protein Family (Hsp40) Member C2; FC = 1.775; *q* = 0.052) and *HSPA1L* (Heat Shock Protein Family A (Hsp70) Member 1 Like; FC = 3.048; *q* = 0.003) [38], upregulated in Mangalitsa and Moravka, respectively. Also, *UBE2V2* (Ubiquitin Conjugating Enzyme E2 V2; FC = 1.763; *q* = 0.094), *USP53* (Ubiquitin Specific Peptidase 53; FC = 2.491; *q* = 0.054) and *USP8* (Ubiquitin Specific Peptidase 8; FC = 1.705; *q* = 0.099) are genes involved in the ubiquitination process, all being overexpressed in the Mangalitsa breed. Finally, *PSMA3* (Proteasome 20S Subunit Alpha 3; FC = 1.901; *q* = 0.035) and *PSMC6* (Proteasome 26S Subunit, ATPase 6; FC = 2.070; *q* = 0.054) were both overexpressed in the Mangalitsa group, being components of proteasomes, which are multicatalytic complexes involved in the degradation of most intracellular ubiquitinated proteins. The proteasome is essential in maintenance of protein homeostasis and cellular function, being responsible of the removal of misfolded or damaged proteins as well as proteins whose functions are no longer required. Thus, protein metabolism was another process altered in our breed comparison. Nine of the ten genes involved in this process were upregulated in Mangalitsa, indicating a higher protein turnover in the Mangalitsa group, which may be related to lower protein deposition and lower muscle development of this breed. This finding is in agreement with previously reported results of transcriptome comparisons between pure Iberian and crossbred pigs [17] and between Basque and Large White pigs [18] where protein metabolism pathways and genes were activated in the breeds with higher fat deposition and lower muscle development (Iberian and Basque).

In Moravka breed results showed upregulation of some key genes coding for transcriptional regulators involved in muscle development and growth, such as different *MYOD1* family members, several *FOS* genes, *STAT3* or *JUNB*, in accordance with the main phenotypic differences between breeds [1,2]. *MYOD1* (Myogenic Differentiation 1, FC = 2.620; *q* = 0.073) is involved in muscle cell differentiation and muscle regeneration [39]. This transcriptional activator induces fibroblasts to differentiate into myoblasts by promoting transcription of muscle-specific target genes, thus leading to muscle development. While the *MYOD* gene was upregulated in the Moravka group, *MYOD* family inhibitors such as *MDFIC* (MyoD Family Inhibitor Domain Containing; FC = 2.103; *q* = 0.072) and *MRLN* (Myoregulin; FC = 6.093; *q* = 0.000009) were overexpressed in the Mangalitsa group. The *FOS* gene family consists of four members: *FOS* (Proto-oncogene c-Fos; FC *=* 5.040; *q* = 0.261), *FOSB* (FosB Proto-Oncogene, AP-1 Transcription Factor Subunit; FC = 6.602; *q* = 0.051), *FOSL1* (FOS Like 1, AP-1 Transcription Factor Subunit; FC = 9.110; *q* = 0.097) and *FOSL2* (FOS Like 2, AP-1 Transcription Factor Subunit; FC = 1.396; *q* = 0.626); *FOSB* and *FOSL1* being significantly upregulated in the Moravka breed. FOS proteins have been implicated as regulators of cell proliferation, differentiation and transformation [40]. *STAT3* (Signal transducer and activator of transcription 3; FC = 1.561; *q* = 0.07) gene codes a protein involved in many cellular functions, including the control of cell growth and division (proliferation), cell movement (migration) and cell death (apoptosis) [41]. This protein is activated through phosphorylation in response to various cytokines and growth factors including IFNs, EGF, IL5, IL6, HGF, LIF and BMP2. In agreement, the *EGFR* gene (Epidermal growth factor receptor) is also activated in Moravka (z = 2.191). Lastly, *JUNB* (JunB Proto-Oncogene, AP-1 Transcription Factor Subunit; FC = 2.989; *q* = 0.009) is a transcription factor involved in regulating gene activity following the primary growth factor response.

### 3.4. Functional Analysis of Breed Effects

The IPA software was used to functionally interpret the biological consequences of the gene expression differences observed. GO results mainly showed a clear enrichment of biological GO terms related to cancer, hyperplasia and development, (Table S3) most of them being activated in Moravka and reflecting a more proliferative status of the muscle tissue in this breed. Likewise, the most significant gene networks detected are those related to organ morphology and development, cell signaling and interaction, and cellular growth and proliferation. In fact, a relevant proportion of DE genes (34 out of 306) are involved in ontology terms associated to organism development (*p*-value range = 1.3 × 10^−2^ − 1.6 × 10^−4^). This reflects a clear enhancement of biological processes associated with tissue development in line with the main phenotypic difference observed between Mangalitsa and Moravka.

The Canonical Pathways prediction tool in IPA software displays the most significant pathways across the entire dataset. Results of this prediction are shown in Table 4. According to this tool the two main canonical pathways affected by the DE genes are oxidative phosphorylation and mitochondrial dysfunction, with 11 DE mitochondrial genes (*COX6C, MT-ATP6, MT-CO2, MT-ND1, MT-ND2, MT-ND3, MT-ND4, NDUFA1, NDUFA5, NDUFB3* and *UQCRB*), all of them upregulated in Mangalitsa, as mentioned previously. Moreover, oxidative phosphorylation is predicted to be clearly activated in Mangalitsa muscle (z-score = −3.317, *p* value = 0.17 × 10^−7^). This result indicates a higher activity of energy production in the form of ATP. In turn, this result is indicative of a higher proportion of oxidative-type muscle fibers in Mangalitsa, which are characterized by a higher quantity of mitochondria and usually associated with increased intramuscular lipid content [42]. Characterization of muscle fiber types has not yet been performed and compared in the two local breeds analyzed here at any level, histological or transcriptomic, but a preponderance of oxidative ones in Mangalitsa would be in agreement with its higher predisposition for fat accumulation, as observed in other local fatty breeds, such as the Iberian [43]. Interestingly, although the Iberian pig has been more deeply studied at the histological and gene expression levels, and higher proportion of oxidative fibers is histologically observed in this local breed, previous transcriptome studies have not reported any activation of oxidative phosphorylation pathway as observed in Mangalitsa [19,20], thus suggesting a stronger metabolic trend for oxidation in the latter. Nevertheless, differences in the transcriptome results obtained in Mangalitsa vs Iberian could be attributed to the age of the animals, as Iberian pig studies involve early developmental stages. Difference in fiber type proportion between Mangalitsa and Moravka is further supported by higher expression of the *MYH4* gene in Moravka (Myosin Heavy Chain 4; FC = 2.565; *q* = 0.024), which is one of the main myosin heavy chains characteristic of fast glycolytic fibers [44]. Also, nitric oxide (NO) is characteristic of glycolytic muscle fibers and is associated with muscle development and growth [45]. We found upregulation of genes involved in NO synthesis and signaling in Moravka (*NOS1*, *ARG2*, *RYR3*), in agreement with the phenotype differences between breeds and the hypothesis of differential muscle fiber pattern between them. The NOS1 gene, the one directly responsible of NO synthesis, has been previously shown to be upregulated in lean vs fat genotypes [19] and involved in growth and fatness regulation in pigs [46]. As explained before, the Moravka breed is an admixed population [5,6] which was created from an extinct indigenous breed crossed with Berkshire and possibly with Yorkshire [2] and this mixed origin could explain the strong differences in muscle metabolism and fiber profile with respect to the autochthonous Mangalitsa breed, which is also derived from the same primitive local breed but without introgression from Asian or improved genotypes [1,6]. 

Interestingly, although the genes coding for the main detoxification enzymes, such as glutathione peroxidase, catalase or superoxide dismutase, which constitute the primary defense systems to manage the prevention of ROS, are not DE between breeds, different defense and repair systems are significantly altered in our dataset of DE genes. Specifically, NRF2-mediated Oxidative Stress Response pathway is affected (*p* value = 0.3 × 10^−2^) with six DE genes (*FMO1, FOSL1, HERPUD1, JUNB, PMF1/PMF1-BGLAP* and *STIP1*). NRF2 is a master regulator of antioxidative responses which induces the expression of different cytoprotective genes and antioxidant target genes [47]. Also, the NER (Nucleotide Excision Repair) pathway is predicted to be significantly activated in Mangalitsa (z-score = −2.00; *p* value = 0.10 × 10^−3^) due to the upregulation in this breed of four key genes (*CDK7, GTF2H5, POLR2K* and *XPA*). The NER pathway is responsible for the removal of bulky DNA lesions induced by UV irradiation, environmental mutagens and certain chemotherapeutic agents [48,49] and has recently been proposed to participate in neutralizing oxidative DNA damage [50]. Also, the EIF2 signaling pathway, which is significantly affected in our dataset (*p* value = 0.7 × 10^−2^), is involved in the integrated stress response (ISR), which is an elaborate signaling pathway present in eukaryotic cells, which is activated in response to a range of environmental and physiological challenges, including oxidative stress [51]. These results suggest that, while normal antioxidant defense mechanisms are more active in Moravka than in Mangalitsa, increased oxidative stress and consequent damage may be occurring in muscle from Mangalitsa pigs, leading to activation of response and repair processes.

Oxidative stress and impairments in antioxidant mechanisms have been reported to be involved in situations of obesity and diabetes [52]. Indeed, reactive oxygen species have been attributed a causal role in insulin resistance and inflammatory processes associated to obesity. Fatty pig breeds, as Mangalitsa, are characterized by a marked trend for fat accumulation and are usually considered adequate animal models to better understand the fattening processes, obesity and metabolic disorders, the Iberian breed being the most studied one [53,54,55]. Although phenotypically the animals studied here do not significantly differ in fattening traits, there is a numerical difference in intramuscular fat percentage, which matches the usual difference found between Mangalitsa and Moravka breeds [1,2] and lack of a clear effect with statistical significance could be a consequence of insufficient biological replication in the present study, as well as difference in slaughter weight between the experimental groups. In agreement, several key molecules and canonical pathways related to fat accumulation are predicted as affected by the differential expression results, such as Leptin signaling in obesity, Adipogenesis pathway or Glucocorticoid receptor signaling (Table 4), which may be related to metabolic differences between breeds in energy homeostasis or antioxidant/inflammatory processes.

Findings regarding the molecular basis of the higher muscle growth observed in Moravka also involve the cAMP-mediated signaling pathway, which is significantly activated in Moravka (z-score = 1.00, *p* value = 0.7 × 10^−2^) and the HIPPO signaling pathway, which is significantly affected (*p* value = 0.9 × 10^−2^). The cAMP-mediated signaling pathway promotes muscle growth and regeneration, contributing to hypertrophy and increased myofiber size [56]. The HIPPO pathway controls tissue growth in many different cell types, being a critical regulator of myogenesis and skeletal muscle mass [57]. The DE genes involved in these relevant pathways are new interesting candidate genes for explaining the variability in growth and carcass traits.

### 3.5. Prediction of Regulators for Breed Effects

**Figure 2:**
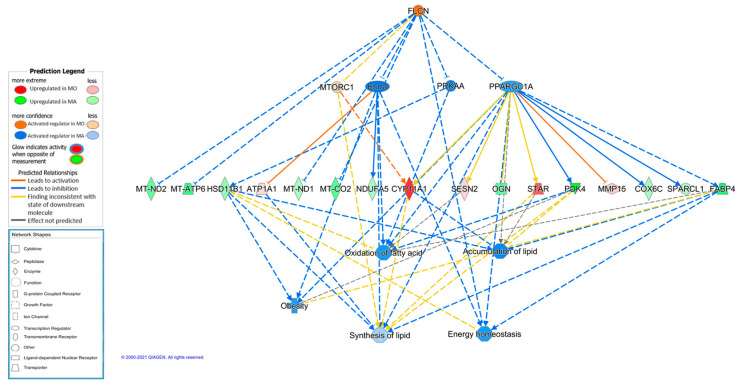
Causal network predicted using Ingenuity Pathway Analysis software where FLCN (folliculin, predicted activated in Moravka) regulates PPARGC1A (PPARG coactivator 1 alpha, inhibited in Moravka) which would increase functions involved in lipid metabolism and energy production in Mangalitsa group.

An in-silico prediction of upstream regulators was performed, in order to identify potential transcriptional regulators which could be involved in the observed differences in gene expression. The dataset of DE genes also allowed the identification of several causal networks involving the predicted regulators and the DE genes. A considerable number of potential regulators were detected, and many were predicted to be activated in each one of the analyzed breeds (Table 5). Among them, several molecules can be highlighted as strong regulatory candidates. For instance, PPARGC1B is predicted to be responsible of the observed changes in mitochondrial and lipid metabolism genes, all being upregulated in Mangalitsa. The regulator is predicted to be activated in Mangalitsa group. This is concordant with the well-established role of PPARGC1B as regulator of FA oxidation, oxidative phosphorylation and mitochondrial biogenesis in muscle [58,59,60]. Moreover, an interesting causal network is proposed to regulate PPARGC1A and downstream mitochondrial and lipid metabolism genes. This causal network is controlled by FLCN (Figure 2) which is predicted to be activated in Moravka, leading to inhibition of downstream genes involved in lipid synthesis and accumulation and energy production, which would be activated in the Mangalitsa breed. It has been shown that FLCN deficiency and subsequent increased *PPARGC1A* expression result in increased mitochondrial function and oxidative metabolism as the source of cellular energy [61].

Several growth factors such as IGF1, EGFR or BMP6, involved in cell proliferation, are potentially mediating the differences in gene expression between breeds, being activated in Moravka. FOXO1 and FOXO3, also predicted as activated in Moravka (z = 2.2 in both cases and *p*-value = 3.2 × 10^−1^ in FOXO1 and *p*-value = 8.7 × 10^−2^ in FOXO3) are transcription factors which have been proposed to play a role in myogenic growth and differentiation and energy metabolism. FOXO1 transcriptional activity is in turn regulated by IGFs and regulates FOS gene expression in skeletal muscle [62]. Interestingly, many of these regulators (MYOD1, FOS, FOXO1 and FOXO3) were predicted to also be involved in muscle transcriptome differences between pure Iberian and crossbred pigs, differing in fat and muscle deposition, at different ages, highlighting their main role in muscle development [63].

**Figure 3:**
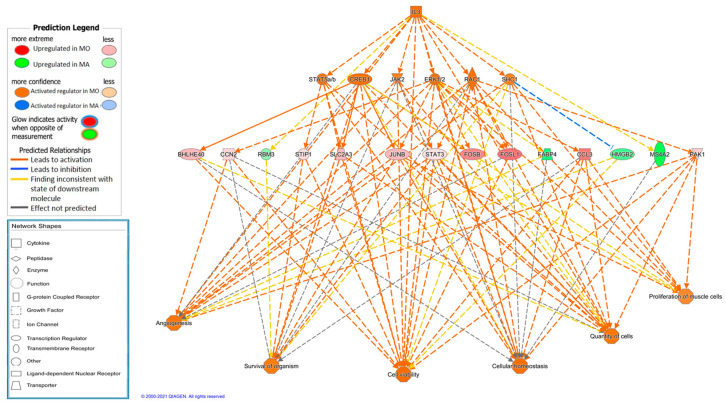
Causal network predicted using Ingenuity Pathway Analysis software with IL3 (interleukin 3) as main regulator and enriched biological functions in Moravka breed.

One of the complex causal networks predicted by IPA (Table S4) is controlled by IL3 (Figure 3). IL3 is a potent growth promoting cytokine, capable of supporting the proliferation of a broad range of cell types. It is involved in a variety of cell activities such as cell growth, differentiation and apoptosis [64].

Several other interesting master regulators are detected such as TRAF2, which has an essential function in muscle development and integrity [65] and regulates a complex and dense causal network activated in Moravka, which involves 12 transcription factors (Jnk, MAP2K1, Map3k7, MAP3K8, MAPK8, NFkB complex, P38 MAPK, RELA, SREBF1, STAT3, STAT5a/b, TRAF2); as well as 26 DE genes. Also, VEGFB (Vascular endothelial growth factor B) is predicted to be the main regulator of a causal network controlling as many as 41 regulators and 72 DE genes, activated in Moravka. VEGFB is a growth factor with an essential role in both vasculogenesis and angiogenesis in skeletal muscle, and is regulated by hypoxia, oxidative stress, growth factors and cytokines [66]. The increase in blood vessel recruitment derived by VEGFs is coupled to muscle growth. The causal network controlled by VEGFB and activated in MO is, in agreement, mainly involved in organismal development (*p* value = 0.2 × 10^−25^; 75 molecules involved) and cellular growth and proliferation (*p* value = 0.2 × 10^−21^; 74 molecules involved) and is significantly enriched in many other relevant functions as organism survival, cell viability, protein synthesis, immune response or skeletal and muscular system development and function.

**Figure 4:**
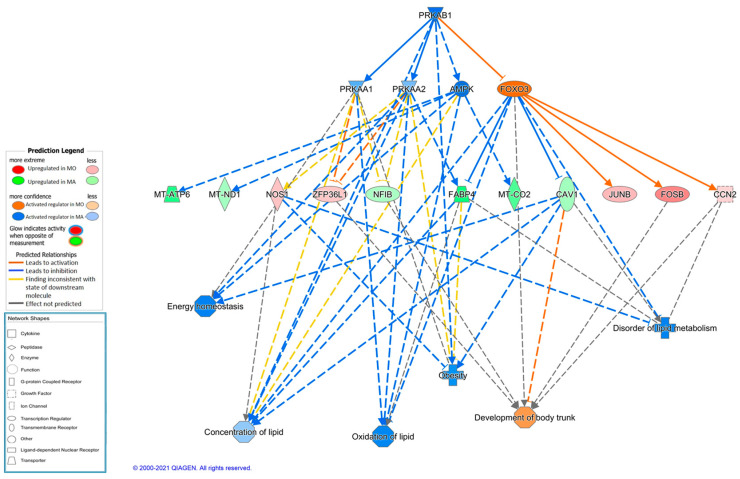
Causal network predicted using Ingenuity Pathway Analysis software where PRKAB1 (protein kinase AMP-activated non-catalytic subunit beta 1) is regulator molecule activated in Mangalitsa breed involved, among others, in lipid metabolism functions.

At last, PRKAB1 was predicted as the key molecule involved in a regulatory causal network activated in Mangalitsa (Figure 4). The protein encoded by this gene is a regulatory subunit of the AMP-activated protein kinase (AMPK), which is an important energy-sensing enzyme that monitors cellular energy status and regulates key enzymes involved in *de novo* biosynthesis of fatty acid and cholesterol [67,68]. Functional prediction of this network is in agreement with an improved development in Moravka, vs lipid metabolism and lipid accumulation issues in Mangalitsa, in agreement with its fatty phenotype and with a potential energy homeostasis unbalance similar to that observed in Iberian pig tissues [69,70].

### 3.6. Tannin Effect on Transcriptome

Tannins are water-soluble polyphenols with either adverse or beneficial effects depending on their concentration and nature [71]. In general, tannin supplementation in animal diets provides antimicrobial, antiparasitic and antioxidant properties but in inadequate amounts and forms it can act as antinutritive substance, showing adverse effects including hepatotoxicity, toxic nephrosis, feed intake depression and growth reduction. Moreover, tannins have bitter or astringent taste contributing to reduced palatability, voluntary feed intake and growth performance. It has also been shown that they inhibit specific gastric, intestine and pancreatic enzymes [72], thus altering absorption of nutrients in intestines [73], reducing protein digestibility [74], and increasing the excretion of endogenous proteins [75]. Nevertheless, pigs from the Mediterranean region, including Iberian and Alentejana breeds, often consume large amounts of acorns (containing 3–7% of tannins) during the final fattening phase in the “montanera” traditional extensive system [76]. This example of positive effects of tannins can be explained by adaptation to high tannin levels through the tannin-binding salivary protein from saliva [77].

On the other hand, feeding weaned piglets with tannin wood extract can improve feed efficiency and reduce intestinal bacterial proteolysis [30], resulting in lower counts of harmful and increased counts of beneficial microorganisms in feces as well as improved growth performance during pre-fattening and fattening periods [78]. Despite the general belief negative impact of tannins on growth performance, Lee et al. [79] suggested that diet of pigs could be supplemented up to 5 % with tannin-rich chestnut. A recent study by Čandek-Potokar et al. [11] demonstrated no negative effects on growth performance in boars supplemented with 1 and 2% extract of hydrolysable tannins. In our work, Mangalitsa animals were supplemented with 1.5% tannins, without negative effects on growth and with positive effect on IMF. Comparison between transcriptomes of these groups showed a small effect, with only 23 genes detected as differentially expressed (Table S2). Out of them, 10 were overexpressed in the control group and 13 in the tannin-supplemented group.

Several genes with relevant roles in developmental processes were upregulated in the control group, such as *TNFRSF12A, DAPK3*, *ARID5A* or *HDAC5*. *TNFRSF12A* (tumor necrosis factor receptor superfamily member 12A, FC = 2.954; *q* = 1.12 × 10^−6^) is a gene involved in angiogenesis, organism growth and apoptosis, which has been shown to be regulated by diet in pig muscle in a linseed supplementation study [80]. *DAPK3* (Death Associated Protein Kinase 3, FC = 2.625; *q* = 3.4 × 10^−4^) gene codes a serine/threonine kinase which is involved in the regulation of apoptosis, autophagy, transcription, translation, actin cytoskeleton reorganization, cell cycle progression and cell proliferation. This gene has been shown to be modulated in mice supplemented with bioactive plant compounds [81]. *ARID5A* (AT-Rich Interaction Domain 5A, FC = 2.805; *q* = 0.087) gene is included in a gene family with diverse functions in development, tissue-specific gene expression and regulation of cell growth. *HDAC5* (Histone Deacetylase 5, FC = 1.550; *q* = 0.099) can influence the critical role of histones in transcriptional regulation, cell cycle progression and developmental events. The upregulation of *HDAC5* in the control group is in agreement with the reported inhibitory role of tannic acid on HDACs [82]. These findings may be related to previously reported anti-proliferative and pro-apoptotic effects of hydrolysable tannins [83].

Among the genes overexpressed in the tannin-supplemented group several interesting candidate genes were found, such as *PPARGC1B* (Peroxisome proliferator-activated receptor gamma coactivator 1-beta, FC = 1.930; *q* = 0.007) gene, which has a coded protein that stimulates the activity of several transcription factors and nuclear receptors, and is involved in fat oxidation, non-oxidative glucose metabolism and the regulation of energy expenditure. This finding may be associated with the higher fat content found in the muscle of supplemented animals. Also, *GRIP2* (Glutamate Receptor Interacting Protein 2, FC = 2.358; *q* = 0.007) gene is induced by tannins, which belongs to a gene family involved in muscle contraction and development [84]. An important paralog of this gene is *GRIP1*, which was significantly associated with backfat thickness traits in pigs [85]. 

Only one DEG was common to both breed and tannin supplementation effects. The gene *TMEM246* (transmembrane protein 246), involved in lipid remodeling, was upregulated in MO and in MAT experimental groups.

### 3.7. Functional Analysis of Tannin Effects

The functional analysis of differential expression results yielded several significantly enriched GO terms, the most significant ones being included in different categories involved in cellular development, cell death and survival and cellular growth and proliferation (Table 6). Nevertheless, results regarding development-related functions were unclear, as different growth/death functions were activated in both experimental groups. Results suggest an activation of lipid metabolism processes in the tannin group, which may be related to a potential higher fat accumulation in muscle following tannin supplementation, in agreement with the observed increase in IMF.

**Figure 5:**
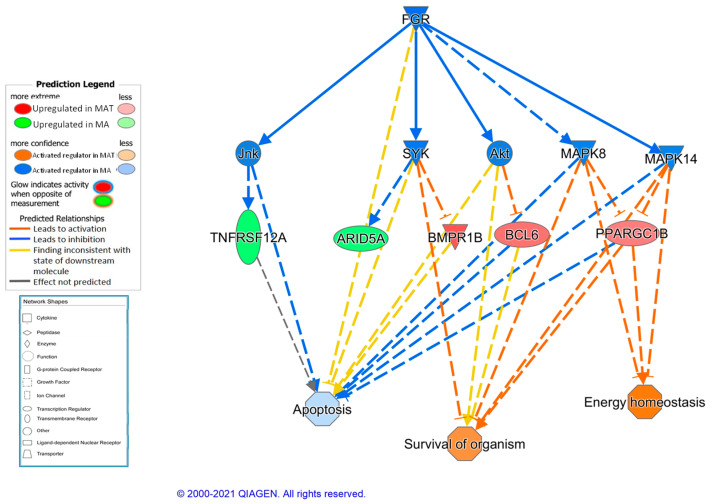
Upstream regulator predicted using Ingenuity Pathway Analysis software, where FGR (FGR proto-oncogene, Src family tyrosine kinase) inhibition leads to activation of survival functions and inhibition of apoptosis in the tannin-supplemented Mangalitsa group.

In spite of the limited number of DEGs, several potential regulators are predicted. The most significant ones are ID2 (*p* value = 0.90 × 10^−4^), a transcription factor which inhibits skeletal myogenesis [86], and EGR2 (*p* value = 0.91 × 10^−4^), involved in skeletal muscle cell differentiation. Also a causal network is predicted as significantly inhibited in the tannin group, which is controlled by the FGR gene (Figure 5) and which may be associated with the activation of cell death processes in the control group vs. activation of performance/survival adaptations in the tannin group. These findings should be interpreted with caution due to the limited number of affected genes and suggestive results but deserve further studies.

## 4. Conclusions

The present study provides the first insights on muscle metabolism regulation in two relevant local Serbian pig breeds at the transcriptome level. Indeed, to our knowledge, no comprehensive gene expression study has ever been previously performed for Mangalitsa and Moravka pigs. A strong effect of the breed was observed on muscle transcriptome, with relevant genes and metabolic pathways being differentially activated in Mangalitsa and Moravka. Although many previous studies have compared muscle transcriptome between breeds with divergent phenotypes, novel findings are reported here, such as the deep regulation in mitochondrial function, oxidative phosphorylation, antioxidant homeostasis and novel pathways potentially involved in muscle development. Also, causal networks and master regulators have been identified, such as FLCN, PPARGC1B or IL3, among many others, which can be considered strong candidate genes to underlie phenotypic variation. Future studies should address the structural variation in these genes, which could provide insight into candidate mutations and regulatory mechanisms supporting the observed gene expression and phenotype differences. A small effect of diet supplementation with hydrolysable tannins on muscle transcriptome was observed in Mangalitsa. This effect was modest in number and magnitude of gene expression differences, with unclear functional interpretation, but suggested effects on relevant metabolic processes such as lipid metabolism, thus highlighting the interest and at the same time the difficulty of the analysis of nutrigenomic effects.

## Figures and Tables

**Table 1:** The composition of feed mixtures.

Ingredients (g/kg)	Mixture I (25–60 kg)	Mixture II (60–120 kg)
Corn (dry)	632.7	691.5
Wheat flour	150.0	150.0
Soybean meal	139.0	90.0
Sunflower meal	50.0	40.0
Calcium carbonate	10.0	9.0
Dicalcium	9.0	10.0
Salt	4.3	4.5
Premix	5.0	5.0
Energy (MJ/kg)	13.6	13.5
Protein (g/kg)	147	130
Lysine (g/kg)	6.6	5.5

**Table 2:** Technical validation of RNA-seq by qPCR: genes, logarithm of fold change (log2 FC) (negative values correspond to upregulation in MA group) and statistical significance obtained with both techniques and Pearson correlation (*r*) between the two used methodologies.

Gene	MA vs. MO					
	RNA-Seq		qPCR		Correlation	
	log2 FC	q	log2 FC	p	r	*p* Value
*ND2*	−1.406	0.019	−1.260	0.0088	0.744	0.013
*ATP6*	−1.661	0.001	−0.980	0.0301	0.626	0.053
*COX2*	−1.497	0.003	−1.407	0.0001	0.724	0.018
*PDK4*	−2.869	0.101	−3.563	0.0157	0.999	2.4 × 10^−12^
*JAZF1*	−1.185	0.065	−0.711	0.0246	0.651	0.041
*NOS1*	1.333	0.010	0.896	0.0091	0.824	0.003
*MYOD1*	1.390	0.074	0.571	0.2939	0.974	1.9 × 10^−6^
*STAT3*	0.642	0.070	0.432	0.1351	0.670	0.076
*FOS*	2.333	0.261	1.102	0.4670	0.877	0.0008
	**MA vs. MAT**					
*ARID5A*	−1.488	0.087	−1.108	0.0028	0.871	0.002
*DAPK3*	−1.392	0.000	−1.447	0.0001	0.894	0.001
*TNFRSF12A*	−1.563	0.000	−1.099	0.0510	0.888	0.001
*RUNX1*	−1.117	0.560	−0.919	0.1590	0.936	0.075
*PPARGC1B*	0.948	0.007	0.503	0.1660	0.620	0.0002
*ACLY*	1.266	0.581	1.088	0.1886	0.992	1.5 × 10^−7^

MA: Mangalitsa; MO: Moravka; MAT: Mangalitsa supplemented with tannins.

**Table 3:** Phenotypic differences between breed and diet groups.

Trait	Least Squares Means			MA vs. MO		MA vs. MAT	
	MA *n* = 12	MO *n* = 10	MAT *n* = 12	SEM	*p*	SEM	*p*
Slaughter Age, days	363.2	357.3	361.9	22.80	0.555	19.63	0.878
Average Daily Gain, g	318.9	366.6	306.4	13.40	0.002	10.22	0.397
Slaughter Weight, kg	115.5	130.2	111.1	5.70	0.018	3.98	0.441
Carcass Weight, kg	89.91	103.04	85.40	4.78	0.013	3.43	0.361
*Longissimus dorsi*							
Loin Thickness, mm	57.5	63.0	54.1	3.0	0.084	1.75	0.182
Intramuscular fat percentage, %	8.55	8.15	11.66	1.56	0.801	0.99	0.037
C14:0, %	2.20	2.18	2.25	0.07	0.787	0.05	0.459
C15:0, %	0.03	0.03	0.03	0.01	0.826	0.00	0.509
C16:0, %	27.92	27.77	28.60	0.50	0.762	0.34	0.166
C16:1, %	3.95	3.22	3.80	0.21	0.002	0.12	0.332
C17:0, %	0.18	0.22	0.16	0.03	0.324	0.02	0.571
C18:0, %	11.09	12.25	11.64	0.33	0.003	0.18	0.044
C18:1, %	46.91	46.53	46.54	0.71	0.597	0.40	0.513
C18:2, %	4.96	4.89	4.40	0.48	0.894	0.31	0.221
C20:0, %	0.18	0.20	0.23	0.01	0.064	0.03	0.343
C18:3n-3, %	0.18	0.19	0.14	0.03	0.720	0.02	0.130
C20:1, %	0.71	0.85	0.71	0.05	0.007	0.02	0.958
C20:2, %	0.36	0.42	0.36	0.04	0.147	0.02	0.872
C20:3n-6, %	0.62	0.67	0.57	0.11	0.603	0.08	0.657
C20:3n-3, %	0.03	0.03	0.07	0.01	0.592	0.04	0.376
C22:1+C20:4, %	0.12	0.11	0.08	0.02	0.422	0.01	0.016

MA: Mangalitsa; MO: Moravka; MAT: Mangalitsa supplemented with tannins; n: number of biological replicates.

**Table 4:** Canonical Pathways derived from Ingenuity Pathway Analysis (IPA) in the set of differential expression genes according to breed comparison.

Ingenuity Canonical Pathways	*p* Value *	Uregulated in MA ^ŧ^	Upregulated in MO ^ŧ^	DE Genes
Oxidative Phosphorylation	0.17 × 10^−7^	**11/109 (10%)**	0/109 (0%)	*COX6C, MT-ATP6, MT-CO2, MT-ND1, MT-ND2, MT-ND3, MT-ND4, NDUFA1, NDUFA5, NDUFB3, UQCRB*
Mitochondrial Dysfunction	0.14 × 10^−5^	11/171 (6%)	0/171 (0%)	*COX6C, MT-ATP6, MT-CO2, MT-ND1, MT-ND2, MT-ND3, MT-ND4, NDUFA1, NDUFA5, NDUFB3, UQCRB*
NER (Nucleotide Excision Repair) Pathway	0.10 × 10^−3^	**4/35 (11%)**	0/35 (0%)	*CDK7, GTF2H5, POLR2K, XPA*
Sirtuin Signaling Pathway	0.14 × 10^−3^	9/291 (3%)	2/291 (1%)	*ARG2, MT-ATP6, MT-ND1, MT-ND2, MT-ND3, MT-ND4, NDUFA1, NDUFA5, NDUFB3, STAT3, XPA*
Estrogen Receptor Signaling	0.27 × 10^−2^	5/328 (2%)	4/328 (1%)	*ARG2, CAV1, MED12, MED6, MMP15, MT-ATP6, PAK1, PLCB4, PLCD1*
NRF2-mediated Oxidative Stress Response	0.36 × 10^−2^	2/189 (1%)	4/189 (2%)	*FMO1, FOSL1, HERPUD1, JUNB, PMF1/PMF1-BGLAP, STIP1*
Wnt/Ca+ pathway	0.45 × 10^−2^	2/62 (3%)	1/62 (2%)	*FZD7, PLCB4, PLCD1*
Protein Ubiquitination Pathway	0.64 × 10^−2^	6/273 (2%)	1/273 (0%)	*DNAJC2, HSPA1L, PSMA3, PSMC6, UBE2V2, USP53, USP8*
EIF2 Signaling	0.69 × 10^−2^	5/223 (2%)	1/223 (0%)	*EIF2S2, EIF3J, EIF5B, RPL22L1, RPL36AL, RPS13*
Leptin Signaling in Obesity	0.70 × 10^−2^	2/74 (3%)	1/74 (1%)	*PLCB4, PLCD1, STAT3*
Glucocorticoid Receptor Signaling	0.70 × 10^−2^	5/336 (1%)	3/336 (1%)	*ANXA1, CCL3, CDK7, GTF2H5, HMGB1, HSPA1L, POLR2K, STAT3*
cAMP-mediated signaling	0.76 × 10^−2^	1/228 (0%)	5/228 (2%)	*AKAP1, CNGB1, GRM2, LAMTOR3, PDE4A, STAT3*
Adipogenesis pathway	0.95 × 10^−2^	3/134 (2%)	1/134 (1%)	*CDK7, FABP4, FZD7, GTF2H5*
HIPPO signaling	0.97 × 10^−2^	0/85 (0%)	3/85 (4%)	*PPP1R10, TEAD3, TP53BP2*

* Significance values (*p*-value of overlap) for the canonical pathways are calculated by the right-tailed Fisher’s Exact Test and indicate the probability of association of molecules from the DE dataset with the canonical pathway by random chance alone. ^ŧ^ Number of genes upregulated in Mangalitsa (MA) and Moravka (MO) in relation to the total number of genes in the pathway. Bold letters indicate significant activation in one breed predicted by IPA.

**Table 5:** Prediction of upstream regulators involved in the gene expression differences between breeds.

Upstream Regulator	Predicted Activation	Activation z-Score	*p*-Value	Target Molecules in Dataset
PDGF BB	Moravka	2.318	2.05 × 10^−4^	*BHLHE40, CCN2, FOSB, JUNB, KLHL21, RYR3, SLC2A3, ZFP36L1*
NFkB	Moravka	2.346	6.85 × 10^−2^	*CCL3, CCN2, DBP, ERAP1, GPR34, HSPA1L, JUNB, MT-CO2, NOS1*
SIRT3	Moravka	2.635	1.31 × 10^−6^	*CYP11A1, MT-ATP6, MT-CO2, MT-ND1, MT-ND2, MT-ND3, MT-ND4*
RICTOR	Moravka	2.121	2.97 × 10^−2^	*NDUFA1, NDUFA5, NDUFB3, POMP, PSMA3, PSMC6, RPS13, UQCRB*
IGF1	Moravka	2.179	1.00 × 10^−1^	*BHLHE40, CYP11A1, FABP4, MYOD1, SLC25A25, STAR*
EGFR	Moravka	2.191	9.58 × 10^−2^	*CAV1, CCN2, HERPUD1, JUNB, MT-CO2, STAT3*
FOXO1	Moravka	2.200	3.20 × 10^−1^	*CAV1, CCN2, FABP4, FOSB, JUNB, PDK4*
MYRF	Moravka	2.000	1.38 × 10^−2^	*CCN2, JUNB, KLHL21, PDK4*
NR4A1	Moravka	2.372	7.78 × 10^−2^	*FABP4, MYOD1, NDUFA1, NDUFB3, PDK4, STAR*
BMP6	Moravka	2.000	3.80 × 10^−4^	*ADAMTS1, CCN2, CYP11A1, FOSL1, STAR, TEF*
FOXO3	Moravka	2.200	8.70 × 10^−2^	*CAV1, CCN2, FABP4, FOSB, JUNB*
ERBB2	Moravka	2.000	3.66 × 10^−1^	*BHLHE40, CCN2, CHST10, JUNB, POLR3A, SMC2, SPARCL1, STAT3*
ALKBH1	Mangalitsa	−2.000	2.12 × 10^−6^	*MT-ATP6, MT-CO2, MT-ND2, MT-ND4*
CAB39L	Mangalitsa	−2.000	1.63 × 10^−3^	*COX6C, NDUFA1, NDUFB3, UQCRB*
NSUN3	Mangalitsa	−2.000	2.12 × 10^−6^	*MT-ATP6, MT-CO2, MT-ND2, MT-ND4*
PPARGC1B	Mangalitsa	−2.236	3.65 × 10^−4^	*MT-ATP6, MT-CO2, MT-ND1, MT-ND2, PDK4*
LONP1	Mangalitsa	−2.646	7.80 × 10^−5^	*GARS1, MT-ATP6, MT-CO2, MT-ND1, MT-ND2, MT-ND3, MT-ND4*
TAL1	Mangalitsa	−2.000	9.89 × 10^−3^	*ARPP21, BCOR, CWC27, FRG1, MS4A2, NOS1, SLC22A16, SLC2A3*
SIRT1	Mangalitsa	−2.201	2.96 × 10^−1^	*CAV1, DBP, EIF2S2, FABP4, PDK4, STAT3*
DAP3	Mangalitsa	−2.449	8.73 × 10^−9^	*MT-ATP6, MT-CO2, MT-ND1, MT-ND2, MT-ND3, MT-ND4*

**Table 6:** Enriched functions identified using Ingenuity Pathway Analysis software in the set of differentially expressed genes, according to diet.

Categories	Functions Annotation	*p*-Value	Predicted Activation	Activation z-Score	Molecules
Cell Death and Survival	Apoptosis	7.49 × 10^−3^	MA	−1.296	*BCL6, BMPR1B, DAPK3, DIO3, HDAC5, PPARGC1B, TNFRSF12A, TNFRSF19, ZBTB16*
Organismal Survival	Organismal death	1.91 × 10^−2^	MA	−1.166	*ARID5A, BCL6, DIO3, HDAC5, KCNAB1, PPARGC1B, TNFRSF12A, ZNRF3*
Cell Death and Survival	Apoptosis of tumor cell lines	8.54 × 10^−3^	MA	−0.588	*BCL6, BMPR1B, DAPK3, HDAC5, PPARGC1B, ZBTB16*
Tissue Morphology	Quantity of cells	4.57 × 10^−2^	MA	−0.532	*ARID5A, BCL6, BMPR1B, DIO3, TNFRSF12A, ZBTB16*
Cellular Development, Connective Tissue Development and Function, Tissue Development	Differentiation of connective tissue cells	1.15 × 10^−4^	MA	−0.045	*ARID5A, BCL6, BMPR1B, HDAC5, PPARGC1B, ZBTB16*
Cellular Development, Cellular Growth and Proliferation	Proliferation of blood cells	2.47 × 10^−2^	MAT	1.887	*BCL6, BMPR1B, TNFRSF12A, ZBTB16*
Cellular Development, Cellular Growth and Proliferation	Colony formation of tumor cell lines	9.51 × 10^−4^	MAT	1.165	*BCL6, HDAC5, PPARGC1B, ZBTB16*
Cellular Development, Connective Tissue Development and Function, Tissue Development	Differentiation of bone cells	8.47 × 10^−4^	MAT	0.988	*BCL6, BMPR1B, HDAC5, PPARGC1B*
Gene Expression	Activation of DNA endogenous promoter	6.12 × 10^−3^	MAT	0.981	*ARID5A, BCL6, BMPR1B, HDAC5, PPARGC1B, ZBTB16*
Lipid Metabolism, Molecular Transport, Small Molecule Biochemistry	Concentration of lipid	4.23 × 10^−3^	MAT	0.638	*DIO3, HDAC5, PPARGC1B, TNFRSF12A, ZBTB16*
Gene Expression	Transcription of RNA	1.05 × 10^−2^	MAT	0.403	*ARID5A, BCL6, BMPR1B, DAPK3, HDAC5, PARGC1B, ZBTB16*
Cellular Development, Cellular Growth and Proliferation	Cell proliferation of tumor cell lines	3.96 × 10^−2^	MAT	0.352	*BCL6, BMPR1B, DAPK3, HDAC5, PPARGC1B, ZBTB16*
Lipid Metabolism, Molecular Transport, Small Molecule Biochemistry	Quantity of steroid	2.87 × 10^−3^	MAT	0.152	*DIO3, PPARGC1B, TNFRSF12A, ZBTB16*

## Data Availability

The results from data analyses performed in this study are included in this article and its tables. Raw sequencing data is available from the corresponding author on reasonable request.

## Supplementary Materials

The following are available online at https://www.mdpi.com/2076-2615/11/3/844/s1, Table S1: Primers details; Table S2: List of differentially expressed genes according to breed and diet; Table S3: Enriched functions identified by IPA in the set of DEGs according to breed; Table S4: List of causal networks detected in the breed comparison.
