# Supplementary material for: Muscle Transcriptome Analysis Reveals Molecular Pathways Related to Oxidative Phosphorylation, Antioxidant Defense, Fatness and Growth in Mangalitsa and Moravka Pigs
Source: Animals (Basel). 2021 Mar 16;11(3):844. doi: 10.3390/ani11030844 (PMC8002519; doi:10.3390/ani11030844)
Supplement: Supplementary file 1 [file animals-11-00844-s001.zip › Table S1 primers details.docx]

| **Gene symbol** | **Gene name** | **Genebank ID** | **Forward primer sequence** | **Reverse primer sequence** | **Efficiency (%)** |
| --- | --- | --- | --- | --- | --- |
| *ACTB* | actin beta | ENSSSCG00000007585 | TCTGGCACCACACCTTCT | TGATCTGGGTCATCTTCTCAC | 90 |
| *B2M* | beta-2-microglobulin | ENSSSCG00000004687 | TTCACACCGCTCCAGTAG | CCAGATACATAGCAGTTCAGG | 88 |
| *GAPDH* | glyceraldehyde-3-phosphate dehydrogenase | ENSSSCG00000000694 | TCGGAGTGAACGGATTTG | CCTGGAAGATGGTGATGG | 97 |
| *ATP6* | ATP synthase F0 subunit 6 | ENSSSCG00000018081 | CTTTTATTGCCCCCACGAT | ATGTTTGTTGAGCCGATGAATA | 94 |
| *COX2* | Cytochrome c oxidase subunit II | ENSSSCG00000018078 | TAAACCTGGAGAAATACGACTACT | ATGGGCATGAAGCTGTGGTTT | 88 |
| *ND2* | NADH dehydrogenase subunit 2 | ENSSSCG00000018069 | ACAAGCCACAGCCTCCATAAT | AGTAGGCCTGCTTGTAGTGAAAT | 95 |
| *MYOD1* | myogenic differentiation 1 | ENSSSCG00000013375 | CGGCGGCGGAACTGCTACG | GGCGCGGCGGGGCTCTCG | 88 |
| *PDK4* | pyruvate dehydrogenase kinase 4 | ENSSSCG00000015334 | AAAGCCCAGAGGATCAGAAAGTAT | TCATCAGCATCCGGGTAGAAA | 95 |
| *FOS* | Fos proto-oncogene, AP-1 transcription factor subunit | ENSSSCG00000002383 | ATCCCAACGGTGACTGCTATCTCG | TGCCCCTTCTGCCAATGCTCT | 90 |
| *NOS1* | nitric oxide synthase 1 | ENSSSCG00000009856 | TGGAGAGGCTGGAGGAGGTGAACA | TGAACATCCCGTGAGCCGTGGTA | 89 |
| *STAT3* | signal transducer and activator of transcription 3 | ENSSSCG00000017403 | AGTGAGTTGGCGGGGCTTTTGT | TCTTAATTTGTTGGCGGGTCTGAA | 95 |
| *JAZF1* | JAZF zinc finger 1 | ENSSSCG00000016691 | CGATTCGGGGGCTGCGGACTC | GGTTGGCTGCTGTAATTCTTGTTT | 91 |
| *DAPK3* | death associated protein kinase 3 | ENSSSCG00000032170 | ACTTCATCCGCCGGCTGCTTGTCA | GGCTTCCGGCTGCTGTCCTCACGA | 92 |
| *TNFRSF12A* | TNF receptor superfamily member 12A | ENSSSCG00000027130 | TGGAGCGCGGACCTAGACAAGTGC | CCAGACCAGGAAGCCAGAAAG | 85 |
| *PPARGC1B* | PPARG coactivator 1 beta | ENSSSCG00000014437 | AGCTCTCCTCCTTCTTCCTCAACT | GCTCGGCGTCGCGGGGGCTGTA | 97 |
| *ARID5A* | AT-rich interaction domain 5A | ENSSSCG00000008123 | AGACAGAAGAAAGCCAAGGAGGAG | GCTAGGTCGGGGGCGTCTGTTTT | 90 |
| *RUNX1* | RUNX Family Transcription Factor 1 | ENSSSCG00000035537 | TGTGATGGCAGGCAATGACGAA | TTGCGGTGGGTTTGTGAAGACG | 93 |
| *ACLY* | ATP citrate lyase | ENSSSCG00000017421 | ATCCGGACCATCGCCATCATC | ATCCCGCCGGTGTTTCCAATCT | 94 |
